# Impact of assisted reproductive technology on the risk of cerebral palsy: A systematic review and meta-analysis

**DOI:** 10.12669/pjms.41.5.11961

**Published:** 2025-05

**Authors:** Xinyu Chen, Peihong Zhou

**Affiliations:** 1 Xinyu Chen Department of Reproduction Eleven Ward, Huzhou Maternity & Child health Care Hospital, Huzhou, Zhejiang Province 313000, P.R. China; 2 Peihong Zhou Department of Gynaecology Thirteen Ward, Huzhou Maternity & Child health Care Hospital, Huzhou, Zhejiang Province 313000, P.R. China

**Keywords:** Cerebral palsy, Assisted reproductive techniques, In vitro fertilization, Intracytoplasmic sperm injection, Multiple pregnancy, Premature birth, Neurodevelopmental disorders

## Abstract

**Objective:**

The aim of this study was to evaluate the impact of assisted reproductive technology (ART) on the risk of cerebral palsy (CP) in offspring.

**Methods:**

We searched PubMed, Embase, Web of Science and Scopus databases for observational studies that investigated the link between ART and CP as a pregnancy outcome that were published until 15th January, 2024. Only studies that provided adjusted effect sizes for the outcome of interest were included. Pooled odds ratio (OR) was reported along with 95% confidence intervals (CI).

**Results:**

Thirteen studies were included. Pregnancy that was achieved with the help of ART correlated with significantly higher risk of CP in offspring (OR 1.51, 95% CI: 1.18, 1.94) compared to natural conception. The risk of CP in singleton pregnancies achieved through ART was elevated (OR 1.31, 95% CI: 1.09, 1.58). while no such increase in the risk was detected in cases of multiple pregnancies (OR 1.07, 95% CI: 0.95, 1.20) and preterm births (OR 1.09, 95% CI: 0.93, 1.28). Studies published prior to the year 2010 showed an association of ART with elevated rates of CP (OR 1.67, 95% CI: 1.14, 2.44). No difference was detected in studies published after the year 2010 (OR 1.40, 95% CI: 0.97, 2.03).

**Conclusion:**

Our findings suggest a modest increase in the risk of CP that is linked to ART. However, the analysis is hindered by a small number of studies and notable heterogeneity. Subgroup analyses did not show significant associations for multiple or preterm pregnancies, suggesting that other factors beyond ART may be responsible. Factors such as maternal age, underlying infertility, and pregnancy complications might contribute to the observed risk of CP rather than ART itself. Higher-quality studies are required to validate our conclusions.

## INTRODUCTION

Cerebral palsy (CP) result from damage to the developing brain.^1^ With a global prevalence of approximately 1.5–3.5 per 1000 live births, CP stands as a leading cause of motor disability in children, imposing a considerable burden on both families and the healthcare system.^2^ Etiology of CP development remains unclear due to its multifactorial and heterogeneous nature, and may be triggered by events that occur during the prenatal, perinatal, and/or postnatal periods.^3^,^4^ Assisted Reproductive Technology (ART) became a pivotal component of infertility treatments.^5^,^6^ The percentage of births resulting from ART procedures ranges between 1% and 4% of all births across countries worldwide i.e., roughly around five million babies.^7^,^8^

While the efficacy of ART in achieving successful pregnancies is well-established, studies suggest its potential association with adverse perinatal outcomes, including preterm delivery, multiple births, low birth weight, birth defects, and intrauterine growth restriction.^9^,^10^ All these factors may contribute to the development of CP in affected children. However, the link between ART and CP risk is still unclear. While some studies show an increased incidence of CP in offspring of ART pregnancies compared to natural pregnancies, others find no such link.^11^–^14^ Studies by Wang et al and Djuwantono et al, have provided valuable insights into the potential association between ART and the risk of CP.^15^,^16^

A review by Wang et al incorporated nine studies and reported a twofold higher risk of CP in children born through ART. However, the extent of the association diminished in subgroup analysis of singleton pregnancies, and became non-significant in cases of multiple pregnancies or preterm births.^15^ Importantly, the review pooled findings from few studies that did not account for potential confounders. Similarly, Djuwantono et al, in their review of five studies, documented a 1.8-times higher risk of CP as an outcome of ART pregnancy compared to natural conception. However, inclusion of studies lacking adjustment for crucial confounders introduced a potential source of bias.^16^

The complex nature of the link between ART and CP is affected by the potential confounding effects of various risk factors, including parental characteristics, care during pregnancy, perinatal care, and adverse birth outcomes.^17^,^18^ The importance of adjusting for these confounders cannot be overstated, as their influence on the observed associations could significantly impact the reliability of conclusions drawn from the studies. This review aimed to critically evaluate available literature to assess the correlation between ART and the risk of CP.

## METHOD

PubMed, Embase, Web of Science and Scopus were screened for relevant studies. The search strategy included relevant terms: (“reproductive techniques, assisted”[MeSH] OR “in vitro fertilization”[MeSH] OR “intracytoplasmic sperm injections”[MeSH]) AND (“cerebral palsy”[MeSH] OR “neuromuscular diseases”[MeSH] OR “motor disorders”[MeSH]). Our interest was to identify studies that were published until 15^th^ January, 2024. Bibliographies of the included studies were also searched to identify additional relevant papers.

### PICOS inclusion criteria:

### Population (P):

Studies including children born through assisted reproductive technology (ART).

### Intervention/Exposure (I):

Conception through any form of ART, including in-vitro fertilization (IVF), intracytoplasmic sperm injection (ICSI), frozen embryo transfer (FET), and ovulation induction.

### Comparator (C):

Naturally conceived children (control group).

### Outcome (O):

Diagnosis of cerebral palsy (CP), with effect estimates adjusted for potential confounders.

### Study Design (S):

Observational studies (cohort and case-control studies) that provide adjusted effect sizes for the association between ART and CP. Only publications in English or with a readily available English translation were considered to ensure clear access to all necessary data for thorough evaluation.

### Exclusion criteria:

Cross-sectional studies, case reports, case series, reviews and conference abstracts, and studies lacking a control group (natural conception) were excluded. In cases of overlapping data, the most recent study was selected for inclusion.

The initial list of studies, identified by the literature search, was deduplicated. Two study authors independently reviewed titles and abstracts of the remaining studies (XC and PZ). A thorough assessment of the full texts of the relevant studies was then conducted to determine their eligibility for inclusion. All disagreements regarding study inclusion were solved through discussions. Two authors independently extracted the data (XC and PZ). For this, a structured form was created, pilot tested, and necessary adaptations were made. The key variables included study identification (author name and year of publication), design and location, databases used for collecting information, sample size, findings on outcome of interest and variables adjusted in the multivariable model.

We relied solely on the data reported in the published studies and did not contact authors for missing information. This approach was taken to ensure transparency and reproducibility of our findings, as all included data were publicly available and accessible to other researchers. The Newcastle-Ottawa Scale (NOS) was used for quality assessment.^19^ The PRISMA guidelines were followed, and the review protocol was registered before starting the work (PROSPERO; registration number: CRD42024507545).^20^

The pooled effect size was reported as odds ratios (OR) and associated 95% confidence intervals (CI). Random effects model was used when there was significant heterogeneity, as indicated by I^2^>50%.^21^ Subgroup analysis was done based on the study design, sample size and year of publication. An assessment of publication bias was done with Egger’s test.^22^ P <0.05 was deemed significant.

## RESULTS

**Fig.1:**
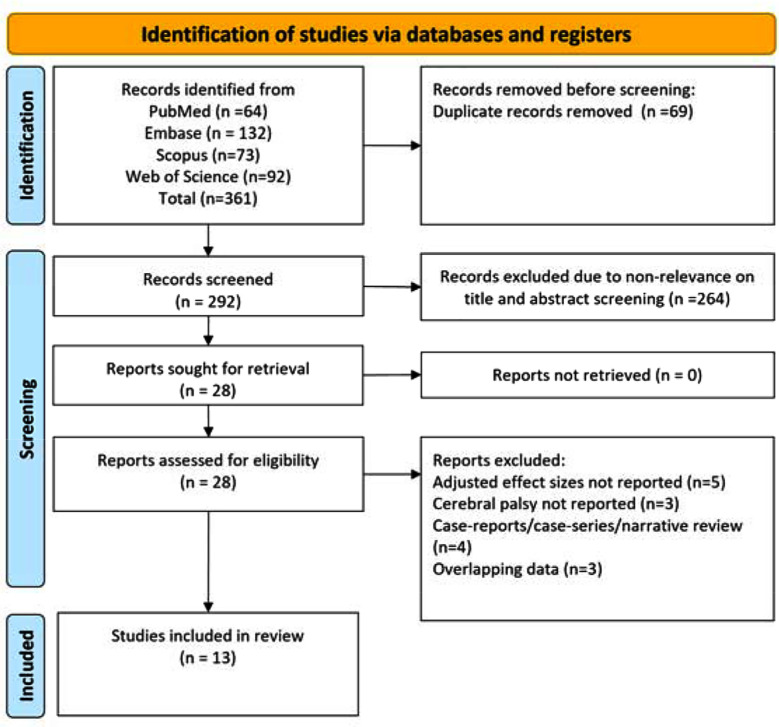
Process involved in selecting the studies for inclusion in this meta-analysis.

A total of 361 studies were identified by the search. Of them, 69 duplicate studies were removed and the screening of title and abstract was done for 292 studies. Based on this screening, 264 studies were excluded. Full texts of the remaining 28 studies were read thoroughly. This resulted in the exclusion of further 15 studies. The final analysis incorporated a total of 13 studies (Fig.1).^11^–^14^,^23^–^31^

Eleven out of thirteen studies had a retrospective cohort design (Table-I). Remaining two studies had a prospective cohort and case-control design, respectively. Four studies were conducted in Australia and two studies each in Denmark and Sweden. One study each was conducted in Norway, Canada, France and Finland. Remaining study was multicentric and conducted in the Nordic countries (Table-I). There were seven studies that were published after 2010, and remaining six studies prior to the year 2010. The sample of children in the included studies ranged from 1473 to 47,91,195. All studies were of good quality. There were seven studies with a score of Eight and six studies with a score of seven (Table-I). All the studies had adjusted for important confounders and that added to their methodological quality.

**Table-I:** Included studies and their key features.

Author	Study design; location	Maternal age (years)	Data source and method for diagnosis of CP	Sample size	Proportion with CP in each group	NOS score	Adjusted for
Carlsen et al (2023)	Retrospective cohort; Norway	Majority aged 25 to 39 years in both groups (>80%); Those <25 years were higher in normal conception (NC), compared to ART group (15% vs. 2%)	Medical birth registry; CP diagnosed using clinical criteria	ART: 23,548 Normal conception, NC: 8,08,444	ART: 0.41% NC: 0.19%	8	Parity, mother’s health before pregnancy (≥1 of following conditions: asthma, chronic hypertension, chronic kidney disease, rheumatoid arthritis, epilepsy, diabetes mellitus) and mother’s age at birth
Verhaeghe et al (2022)	Prospective cohort; France	Mean age 32 years (ART group) and 29.4 years (NC group)	Prospective, national cohort study “EPIPAGE-2” CP diagnosed using clinical criteria	ART: 814 NC: 3535	ART: 4.5% NC: 5.1%	7	Gestational age, antenatal steroids, maternal age, parity, education level, employment status, living with a partner, smoking during pregnancy, country of birth, and parents’ socioeconomic status
Roychoudhury et al (2021)	Retrospective cohort; Canada	Mean age 34.6 years (ART group) and 30.8 years (NC group)	Canadian Neonatal Network (CNN) database CP diagnosed using clinical criteria	ART: 651 NC: 4212	ART: 4.6% NC: 5.6%	8	Gestational age, antenatal steroids, sex, small for gestational age, multiple gestations, caesarean delivery, maternal age, maternal education, hypertension, diabetes mellitus, or smoking. Generalized estimating equations model clustered by twin pairs
Spangmose et al (2021)	Retrospective cohort; Multicentric (Nordic countries)	Mean age 33.9 years (ART group) and 30.0 years (NC group)	National Medical Birth Registers (CoNARTaS cohort including all deliveries in Denmark, Finland, and Sweden) CP based on ICD-9 or 10 codes	ART: 1,11,844 NC: 467,9,351	ART: 5.9% NC: 3.5%	8	Parity, child’s sex, country, maternal age and maternal smoking during pregnancy, maternal educational level, preterm birth and low birth weight.
Goldsmith et al (2018)	Retrospective cohort; Australia	Mean age 34.5 years (ART group) and 29.3 years (NC group)	The statutory Midwives’ Notification of Births System records data; the Reproductive Technology Register; the Western Australian Register of Developmental Anomalies Diagnosis of CP based on medical records	ART: 2914 NC: 2,08,746	ART: 0.72% NC: 0.25%	7	Year of birth, maternal age, and parity.
Abdel-Latif et al (2013)	Retrospective cohort; Australia	Mean age 33.0 years (ART group) and 30.0 years (NC group)	10 neonatal intensive care units (NICUs) Diagnosis of CP based on clinical assessment	ART: 217 NC: 1256	ART: 9.2% NC: 8.8%	8	Maternal age, multiple pregnancy, gender, gestational age and birth weight
Davies et al (2012)	Retrospective cohort; Australia	Proportion of mothers aged 20-24 years higher in NC group, compared to ART group (20.8% vs. 2.2%) Proportion of mothers aged over 35 years higher in ART group, compared to NC group (31% vs. 12%)	Data from two fertility clinics Diagnosis of CP based on ICD-9 codes	ART: 6163 NC: 3,02,811	ART: 0.4% NC: 0.2%	8	Maternal age, parity, sex, year of birth, maternal race or ethnic group, maternal country of birth, maternal conditions in pregnancy, maternal smoking during pregnancy, socioeconomic status, and maternal and paternal occupation
Hvidtjørn et al (2010)	Retrospective cohort; Denmark	Proportion of mothers aged 20-34 years higher in NC group, compared to ART group (86.2% vs. 71.8%)	Danish national registers Diagnosis of CP based on medical records	ART: 33,139 NC: 5,55,827	Data not provided	7	Sex, maternal age, education, smoking and parity
Källén et al (2010)	Retrospective cohort; Sweden	Group-wise data on maternal age not provided	Swedish Medical Birth Register and Swedish Patient Register Diagnosis of CP based on hospital discharge records (ICD-9 based)	ART: 31,587 NC: 2,591,930	ART: 0.44% NC: 0.24%	7	Year of birth, maternal age, parity, and smoking
Reid et al (2010)	Case-control; Australia	Majority of the mothers (>85%) were aged 20 to 39 years in both groups	Victorian Cerebral Palsy Register, the Victorian Perinatal Data Collection Unit, and three IVF centres in Victoria Diagnosis of CP based on medical records	ART: 41 NC: 3682	Not applicable as it’s a case-control study	7	Parity, sex, and history of recurrent miscarriages, gestational age and birthweight
Zhu et al (2010)	Retrospective cohort; Denmark	Data not provided	Danish National Birth Cohort Diagnosis of CP based on clinical examination	ART: 3000 NC: 13462	ART: 0.57% NC: 0.16%	7	Maternal age, parity, smoking, education, sex of child, multiplicity and preterm birth
Klemetti et al (2006)	Retrospective cohort; Finland	Mean age 33.9 years (ART group) and 29.7 years (NC group)	Finnish medical birth register Diagnosis of CP based on ICD-10 codes	ART: 4527 NC: 26,877	ART: 0.38% NC: 0.14%	8	Socio-economic position of the mother (upper white-collar workers, lower white-collar workers, blue-collar workers, others)
Strömberg et al (2002)	Retrospective cohort; Sweden	Not provided	The National Board of Health and Welfare records Diagnosis of CP based on ICD-10 codes	ART: 5680 NC: 11,360	ART: 0.55% NC: 0.15%	8	Maternal age, year of birth, birth hospital, sex, birthweight and gestational age

### Risk of cerebral palsy (CP):

**Fig.2:**
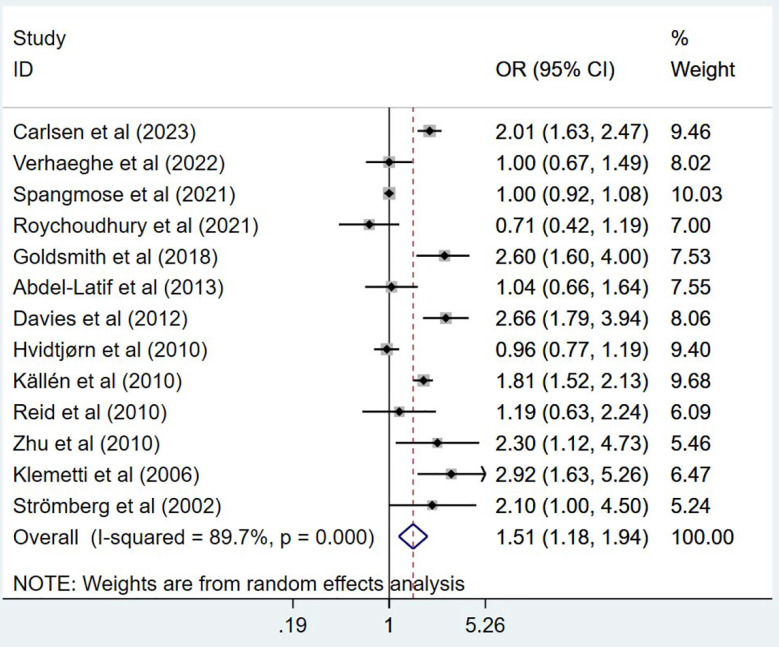
Overall risk of cerebral palsy in those undergoing ART, compared to those conceived naturally.

**Fig.3:**
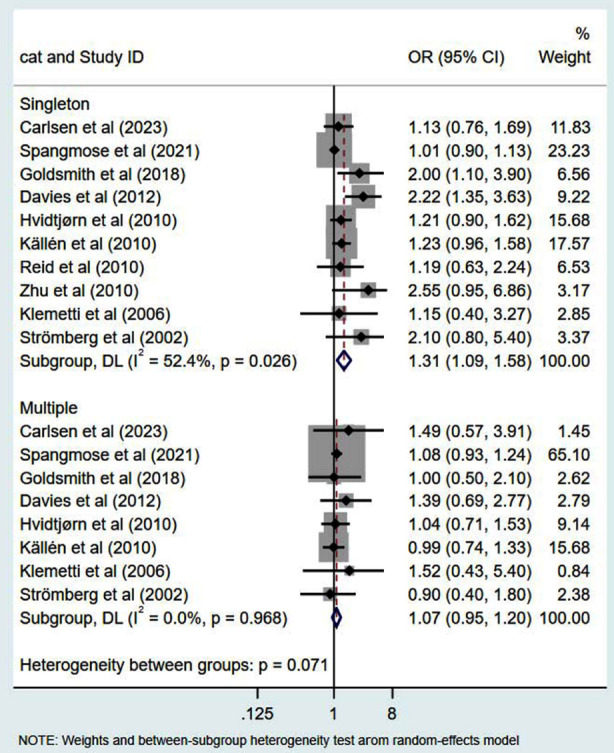
Risk of cerebral palsy in subgroups of singletons and multiples born through ART.

**Fig.4:**
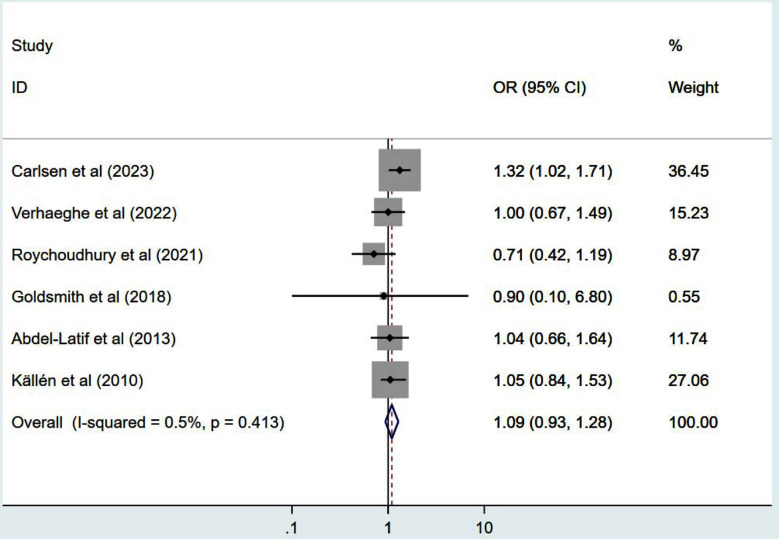
Risk of cerebral palsy in preterms born through ART, compared to preterms conceived naturally.

In comparison to children conceived naturally, children conceived via ART exhibited a higher risk of CP, with OR of 1.51 (95% CI: 1.18, 1.94; n=13, I^2^=89.7%) (Fig.2). The risk for CP in ART-conceived singleton pregnancies was higher compared to natural singleton pregnancies (OR 1.31, 95% CI: 1.09, 1.58; n=10, I^2^=52.4%) (Fig.3). Conversely, the risk of CP was comparable in both groups in cases of multiple pregnancy (OR 1.07, 95% CI: 0.95, 1.20; n=8, I^2^=0.0%) (Fig.3). Additionally, pooled OR for CP risk in preterm children born after ART did not show a significant increase (OR 1.09, 95% CI: 0.93, 1.28; n=6, I^2^=0.5%) (Fig.4). Publication bias was not evident for any of these analyses except for ART singletons (Egger’s p value of 0.006).

Findings of the subgroup analysis indicated that the overall increased risk of CP among children born through ART as well the increased risk in ART singleton pregnancies was observed when studies with a retrospective cohort design, those published prior to the year 2010 and those with larger sample size of 5000 or more were pooled together (Table-II). Subgroup analyses did not detect correlation of ART and CP risk in offspring of multiple pregnancies and in children born preterm (Table-II).

**Table-II:** Subgroup analysis.

Subgroups	Overall risk of CP	Risk of CP in singleton birth	Risk of CP in multiple births	Risk of CP in preterm births
	Pooled odds ratio (OR) with 95% CI (Number of studies; I^2^)	Pooled odds ratio (OR) with 95% CI (Number of studies; I^2^)	Pooled odds ratio (OR) with 95% CI (Number of studies; I^2^)	Pooled odds ratio (OR) with 95% CI (Number of studies; I^2^)
*Study design* Retrospective cohort	1.60 (1.22, 2.11) (11; 91.3%) *	1.33 (1.09, 1.63) (9; 57.6%) *	1.07 (0.95, 1.20) (8; 0.0%)	1.11 (0.94, 1.31) (5; 16.8%)
*Year of study* After year 2010 Prior to year 2010	1.40 (0.97, 2.03) (7; 91.8%) 1.67 (1.14, 2.44) (6; 82.5%) *	1.40 (0.95, 2.08) (4; 77.2%) 1.27 (1.07, 1.51) (6; 0.0%) *	1.09 (0.95, 1.26) (4; 0.0%) 1.01 (0.81, 1.26) (4; 0.0%)	1.11 (0.92, 1.33) (5; 18.9%) 1.05 (0.78, 1.42) (1; ---)
*Sample size* <5000 ≥5000	0.96 (0.76, 1.23) (4; 0.0%) 1.82 (1.34, 2.48) (9; 92.7%) *	1.19 (0.63, 2.24) (1; ---) 1.33 (1.09, 1.63) (9; 57.6%) *	--- 1.07 (0.95, 1.20) (8; 0.0%)	0.93 (0.72, 1.21) (3; 0.0%) 1.19 (0.98, 1.45) (3; 0.0%)

* indicates statistical significance at p<0.05.

## DISCUSSION

Our findings indicate an increased risk of CP in children born through ART, when compared to naturally conceived children. Notably, our results indicated that the elevated risk of CP in ART-conceived children was evident only in studies published before 2010. This might suggest potential influences from evolving ART techniques, changes in clinical practices, and improvements in perinatal care over time. It’s worth noting that the effect size of the association observed in our analysis (OR of 1.5), is smaller than that reported in previous meta-analyses (OR of around 2.0). One plausible explanation for this difference lies in the methodology of previous reviews, where inclusion criteria allowed inclusion of studies lacking adjustment for confounding variables.^15^,^16^

Our analysis of singleton pregnancies that were achieved through ART showed a 30% increased risk of CP. Intriguingly, this elevated risk was not seen in offspring of multiple gestations. These findings align with previous meta-analyses, reinforcing the consistency of our results.^15^,^16^ The underlying aetiology of elevated CP risk is complex and not fully clarified, likely stemming from multifactorial origins.^3^,^4^ Potential reasons include possible subfertility or infertility, which prompts couples to turn to ART, as well as the ART procedures themselves. Infertility is considered a possible contributor to the elevated CP risk associated with ART.^5^,^32^,^33^ Interestingly, increased CP risk has been reported even in offspring of women with infertility who conceive without ART.^29^,^34^ However, further studies are needed to explore this effect.

Multiple gestations are acknowledged as a significant risk factor for CP following ART procedures.^35^,^36^ However, our results do not detect higher risk of CP in offspring of ART-conceived multiple pregnancies. It may be possible that ART itself does not independently elevate the risk of CP in the context of multiple gestations and any observed increased risk is likely attributed to the inherent risks associated with carrying multiple fetuses, such as prematurity, fetal growth restrictions, and zygosity.^37^–^39^

It is crucial to emphasize that many studies often neglect to consider zygosity, despite the established association between monozygosity and an increased risk of CP.^40^,^41^ No discernible association was found between ART and CP in premature births. This finding strengthens the theory that ART does not independently elevate the risk of CP, given that preterm births, which inherently carry a risk of CP, were already considered in this subgroup analysis.^42^,^43^ Prematurity is acknowledged as one of the established causes of long-term neurological disability and is associated with ART procedures.^42^–^44^

Our findings suggest that while ART is associated with an increased risk of cerebral palsy, this risk may be largely influenced by underlying maternal and paternal characteristics rather than ART itself. Individuals undergoing ART often have pre-existing conditions such as advanced maternal age, polycystic ovary syndrome (PCOS), endometriosis, or male factor infertility, all of which have been independently linked to adverse perinatal outcomes, including preterm birth and low birth weight. These conditions, rather than the ART procedure per se, may contribute to the observed association with CP. Moreover, our subgroup analysis suggests that multiple pregnancies and preterm births, common consequences of ART, were not significantly associated with CP risk, raising the possibility that other underlying confounding factors, such as maternal health conditions and reasons for infertility, may be driving this association.

Given these findings, it is important to recognize that ART-conceived children are not inherently at higher risk of CP due to ART itself, but rather due to the complex interplay of infertility-related factors and pregnancy complications. Future studies should focus on disentangling the effects of ART from the inherent risks carried by individuals seeking fertility treatments, particularly by examining more homogenous populations with well-characterized infertility causes.

An additional noteworthy finding in our study is that studies published after the year 2010 did not demonstrate an increased risk of CP in ART pregnancies, in contrast to studies conducted before 2010. This temporal disparity may be linked to the evolving landscape of reproductive practices, such as adopting a single embryo transfer strategy, and more effective implementation of reproductive policies.^45^,^46^ This shift in approach could contribute to a reduction in the occurrence of multiple gestations and, subsequently, mitigate the inherent risks that are associated with them. Additionally, recent advancements in reproductive technologies allow to obtaining embryos of higher quality. This ultimately leads to increased likelihood of achieving successful pregnancies and better neonatal outcomes.^47^,^48^ The combination of refined clinical practices, a more strategic approach to embryo transfer, and technological enhancements could collectively account for the diminishing risk of CP in children who were conceived via ART that was observed in studies published post-2010.

### Limitations:

These must be considered in interpreting the findings of our study. Firstly, there was notable heterogeneity in the outcomes, most likely due to variations in study population characteristics, varied ART methods employed, and the presence of unmeasured confounders. Second, the inability to explore the risk associated with each specific type of ART posed a challenge, as most studies did not provide detailed information on the specific technique employed, grouping various methods under the umbrella term of ART.

Third, the lack of data stratified by maternal age, or medical conditions limited our ability to conduct analyses in these critical subgroups. Fourth, the predominant representation of studies from Nordic countries limits the generalizability of the findings to diverse populations. Fifth, the pathophysiological mechanisms of the link between CP and ART could not be explored. Additionally, control groups in many studies contain children born to normally fertile women with natural conceptions, and therefore may not fully account for the potential influence of subfertility on adverse birth outcomes.

## CONCLUSION

Our study indicates a modest increase in risk of CP associated with ART, potentially linked to elevated rates of multiple gestation and preterm births. However, caution is warranted in interpreting our findings, due to the small number of the included studies and a significant heterogeneity. Further high-quality studies are needed to better understand the strength of the association and explore the underlying mechanisms contributing to higher incidence of CP after ART.

### Authors’ Contributions:

**XC and PZ:** Study design, literature search, data collection, data analysis, interpretation, manuscript writing and Critical review.

**XC:** Manuscript revision and validation and critical analysis.

All authors have read, approved the final manuscript and are accountable for the integrity of the study.
